# Trends and hotspots in non-motor symptoms of Parkinson’s disease: a 10-year bibliometric analysis

**DOI:** 10.3389/fnagi.2024.1335550

**Published:** 2024-01-17

**Authors:** Xuefeng Li, Chunhai Chen, Ting Pan, Xue Zhou, Xiaozhou Sun, Ziyang Zhang, Dalong Wu, Xinhua Chen

**Affiliations:** ^1^Changchun University of Chinese Medicine, Changchun, China; ^2^The Affiliated Hospital to Changchun University of Chinese Medicine, Changchun, China; ^3^Center of Children's Clinic, The Affiliated Hospital to Changchun University of Chinese Medicine, Changchun, China

**Keywords:** Parkinson’s disease, non-motor symptom, global research trend, bibliometrics, visualized study

## Abstract

Non-motor symptoms are prevalent among individuals with Parkinson’s disease (PD) and seriously affect patient quality of life, even more so than motor symptoms. In the past decade, an increasing number of studies have investigated non-motor symptoms in PD. The present study aimed to comprehensively analyze the global literature, trends, and hotspots of research investigating non-motor symptoms in PD through bibliometric methods. Studies addressing non-motor symptoms in the Web of Science Core Collection (WoSCC), published between January 2013 and December 2022, were retrieved. Bibliometric methods, including the R package “Bibliometrix,” VOS viewer, and CiteSpace software, were used to investigate and visualize parameters, including yearly publications, country/region, institution, and authors, to collate and quantify information. Analysis of keywords and co-cited references explored trends and hotspots. There was a significant increase in the number of publications addressing the non-motor symptoms of PD, with a total of 3,521 articles retrieved. The United States was ranked first in terms of publications (*n* = 763) and citations (*n* = 11,269), maintaining its leadership position among all countries. King’s College London (United Kingdom) was the most active institution among all publications (*n* = 133) and K Ray Chaudhuri was the author with the most publications (*n* = 131). *Parkinsonism & Related Disorders* published the most articles, while *Movement Disorders* was the most cited journal. Reference explosions have shown that early diagnosis, biomarkers, novel magnetic resonance imaging techniques, and deep brain stimulation have become research “hotspots” in recent years. Keyword clustering revealed that alpha-synuclein is the largest cluster for PD. The keyword heatmap revealed that non-motor symptoms appeared most frequently (*n* = 1,104), followed by quality of life (*n* = 502), dementia (*n* = 403), and depression (*n* = 397). Results of the present study provide an objective, comprehensive, and systematic analysis of these publications, and identifies trends and “hot” developments in this field of research. This work will inform investigators worldwide to help them conduct further research and develop new therapies.

## Introduction

Parkinson’s disease (PD) is a progressive neurodegenerative disorder involving multiple neurotransmitter pathways in the brain and autonomic nervous system, and is associated with a range of clinical features (Berardelli et al., 2013). Neurotransmitter deficits are known to cause various motor (Postuma et al., 2015) and non-motor symptoms (Li et al., 2021). Non-motor symptoms may occur in individuals ≥20 years of age (Barnett, 2016) and are characterized by an impaired sense of smell (olfactory loss and hyposmia), cognitive deficits, depression, and rapid eye movement (REM) sleep behavior disorders (LeWitt and Chaudhuri, 2020). Alternatively, these and other non-motor symptoms may appear later in the disease course (Titova and Chaudhuri, 2018). For example, as the disease progresses, individuals with PD are more likely to develop mild cognitive impairment, which is an important predictor of progressive dementia (Aarsland et al., 2017; Nicoletti et al., 2019). Non-motor symptoms are a major determinant of quality of life, progressive disability, and dependence in patients with PD, incurring higher medical costs during treatment than those treated for motor symptoms (Leite Silva et al., 2023). Despite their clinical importance, however, non-motor symptoms are often overlooked (Todorova et al., 2014). Identifying non-motor symptoms associated with specific motor subtypes will aid clinicians in anticipating and treating these symptoms.

Over the past few years, the study of non-motor symptoms in PD has progressed, and the volume of scientific literature generated in this field has substantially increased. With this large volume of publications, traditional methods of analysis are no longer sufficient to accurately capture ongoing developments in this area of research. Bibliometrics is an important academic field that focuses on assessing the quantitative attributes, trends, and scholarly impact of the scientific literature (Chen and Song, 2019; Yeung et al., 2021; Yin et al., 2022). By analyzing publications using literature retrieval systems and metrology, researchers can gain a comprehensive understanding of publications from multiple perspectives. This enables them to assess the history, current status, and future potential of these publications as well as their quantity and quality. Through bibliometric analyses, web-based knowledge maps can be drawn, new trends predicted, and recent advances in specific fields observed (Guler et al., 2016; Yin et al., 2021). Bibliometrics is currently used to assess research trends and “hot spots” in various fields, such as disease treatment, pain management, molecular biology, environmental pollution, food safety, and new materials (Chu et al., 2022; Li et al., 2022; Dirpan et al., 2023; Guo et al., 2023; Li C. et al., 2023; Zhang et al., 2023).

In recent years, many bibliometric studies have been published in the biological sector, including those addressing PD movement disorders (Li Y. et al., 2023), however, there is a lack of bibliometric analyses of non-motor symptoms in PD. As such, the present study analyzed the literature addressing non-motor symptoms in the past 10 years using two bibliometric software programs, VOSviewer and CiteSpace, to assess the current status and trends in non-motor symptom PD research globally, which will be divided into two parts. The first is a multidimensional presentation of the past 10 years of research in the field through bibliometric methods, including an assessment of the number and quality of publications globally, as well as bibliographic coupling, co-authorship, co-citation, and co-occurrence analyses, to assess current research hotspots and future research trends in this field. In the second, the bibliometric results were analyzed and summarized to lay the foundation for future research directions on the global non-motor symptoms of PD.

## Materials and methods

### Data source and search strategies

**Figure 1:**
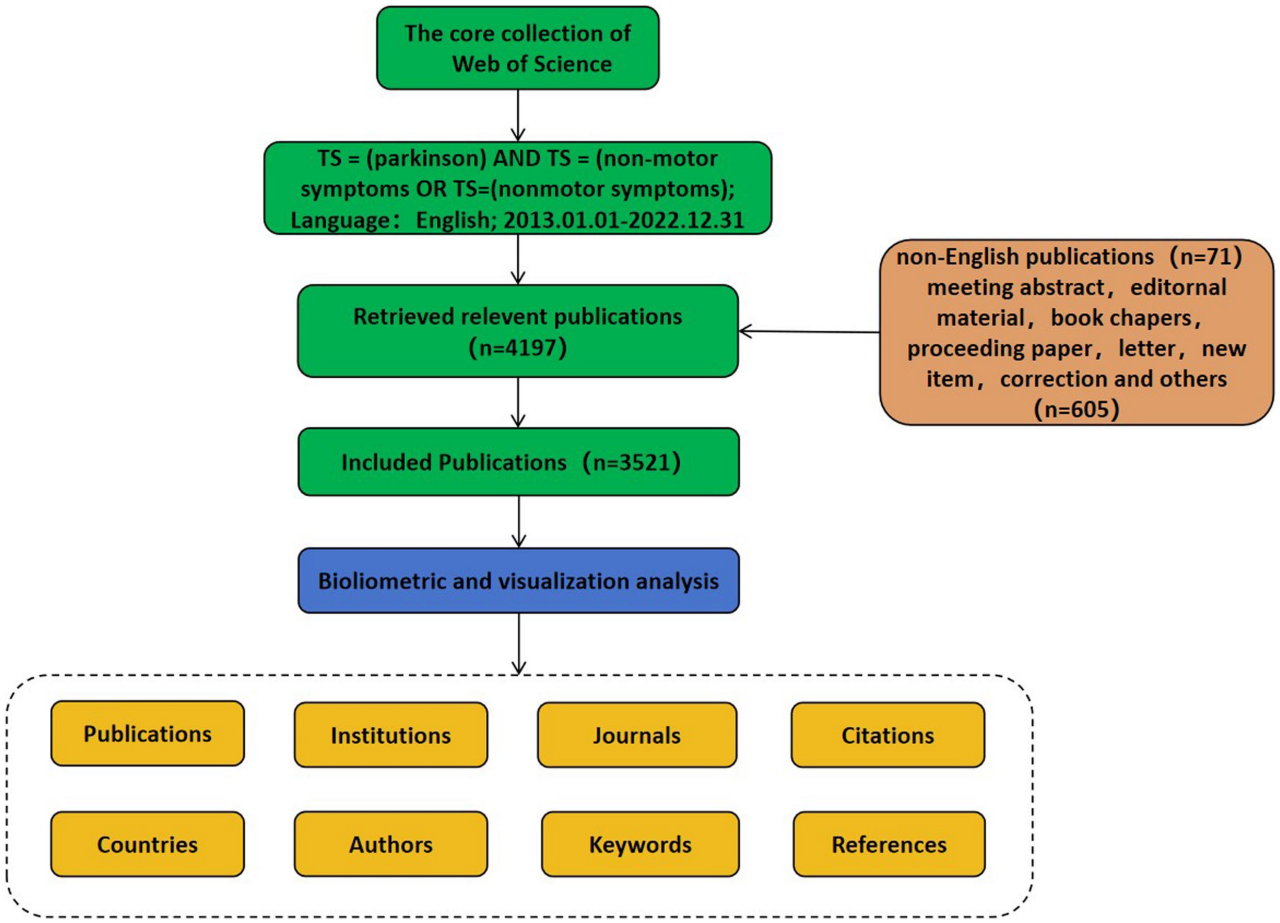
Flowchart of the selection process for the eligible literature.

The Web of Science Core Collection (WoSCC) is a functionally rich database of high-quality digital bibliographic resources with sufficient bibliometric indicators covering published studies in many areas of science, biomedicine, engineering, and technology, and was used to facilitate the analysis (Sevinc, 2004). In this study, Science Citation Index Expanded (SCIE) and Social Science Citation Index (SSCI) were selected to obtain the most comprehensive and accurate studies investigating non-motor symptoms of PD. The following search strategy was used: TS = (Parkinson’s disease) AND TS = (non-motor symptoms) OR TS = (nonmotor symptoms). Search parameters included publication type (articles), language (English), and publication period (January 1, 2013 to December 31, 2022). The literature search retrieved 3,521 articles (Figure 1). All publication searches and literature downloads were performed on August 16, 2023 to avoid bias in continuous database updates. Two researchers independently verified data collection and input. Differences between the results obtained by the two researchers were resolved by consensus discussion(s) or consultation with experts in the field. A complete record of each publication, including title, year of publication, author name, nationality, affiliation, journal name, keywords, and abstract, was used for data analysis.

### Bibliometric analysis

Researchers have created and used bibliometric mapping software. In this study, CiteSpace version 6.2. R4 (Chen, 2004) and VOSviewer (1.6.16) (van Eck and Waltman, 2010) were used to analyze reference co-citations, keyword co-occurrences, keyword outbreaks, and collaborations between countries, institutions, and authors. In addition, spreadsheet software (Excel, Microsoft Corporation, Redmond, WA, United States) was used to collate the data features and plot the number of publications, top 10 institutions, and authors. The Journal Citation Report (JCR), an authoritative multidisciplinary journal evaluation tool, is an important indicator of scientific research (Andersen et al., 2006). The H-index also provides an accurate measure of authors’ scholarly achievements (Hirsch, 2007). JCR classifications and impact factors (IFs) for journals in 2023 were obtained, as well as H-indexes for countries, institutions, and researchers through the Web of Science database. The “Bibliometrix” package of R software was used for data analysis and graphical plotting, Scimago Graphica (Hassan-Montero et al., 2022)^1^ for global geo-visualization of publications, and ChiPlot (Huang H. T. et al., 2023)^2^ for plotting the distribution of keyword years.

## Result

### Temporal distribution of publications and citations

**Figure 2:**
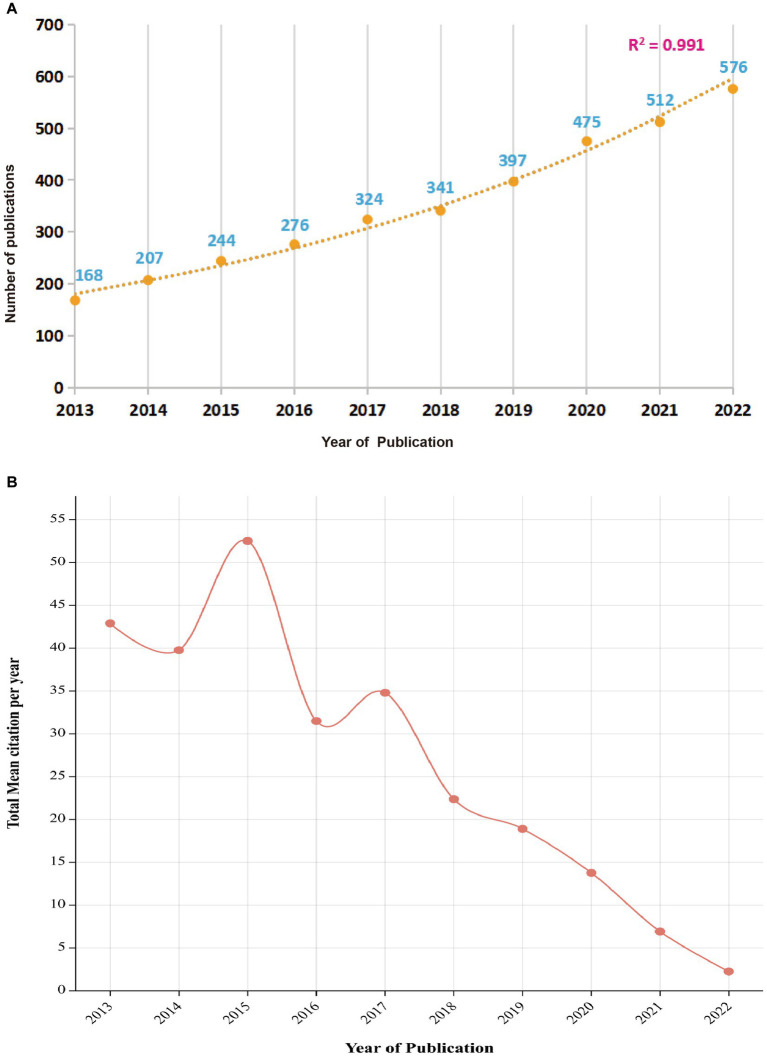
Annual trend chart of publications and citations between 2013 and 2022. **(A)** Total annual number of publications; **(B)** Average annual number of citations.

The literature addressing non-motor symptoms in PD was searched in the WoSCC database for the past 10 years (2013 to 2022), article type was “article” and language was English, with a total of 3,521 publications fulfilling the inclusion criteria retrieved. There has been a steady increase in the number of articles addressing non-motor symptoms of PD over the past 10 years, with an overall upward trend. The predictive model for the trends in the number of articles (*R*^2^ = 0.991) (Figure 2A) suggests that current research investigating non-motor symptoms of PD are gradually attracting the attention of international researchers and may become a potential research “hotspot” in the future. The total mean number of citations per year represents the yearly average number of times each article addressing non-motor symptoms in PD was cited. This metric reveals the overall academic impact of articles published in a given year. The annual mean number of citations for articles addressing PD non-motor symptoms has been decreasing each year as the number of publications has increased rapidly (Figure 2B). However, in 2015 and 2017, there was an upward trend in the average annual number of citations, indicating higher-quality publications.

### Analysing the most productive countries and international cooperation

**Figure 3:**
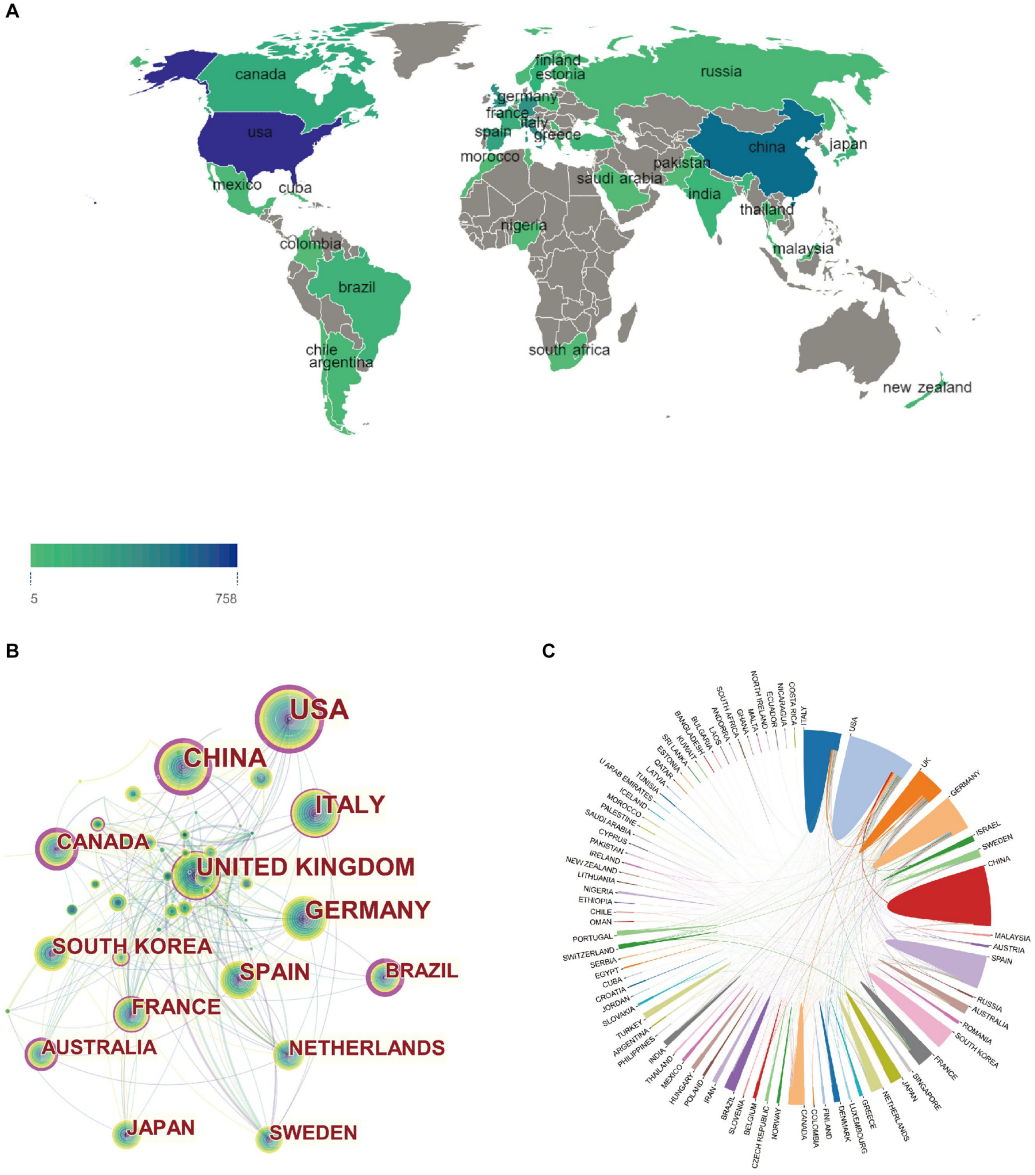
Geographical distribution and network of countries/regions in non-motor symptoms of PD. **(A)** Geographical map of publications; **(B)** A co-occurrence network of countries/regions; and **(C)** A network diagram showing international cooperation.

A total of 84 countries or territories conducted studies investigating the non-motor symptoms of PD between 2013 and 2022. The top 10 countries according to number of publications are listed in Table 1. The United States ranked first among countries that published >200 articles addressing the non-motor symptoms of PD, with 763 (21.7%), followed by China [*n* = 586 (16.6%)], Italy [*n* = 402 (11.4%)], the United Kingdom [UK; *n* = 387 (11%)], Germany [*n* = 386 (10.9%)], and Spain (*n* = 269 [7.6%]). The United States had the highest number of citations (*n* = 11,269), while Canada had the highest mean number of citations (*n* = 53.5), suggesting that the quality of publications from Canada was higher. A geographical map depicting the number of publications according to country is presented in Figure 3A. The national and regional collaborative networks used CiteSpace (Figure 3B), with each node representing a country. Nodes with purple rings represent high centrality (centrality ≥0.1), indicating that they are considered highly important and influential (Synnestvedt et al., 2005). Countries, including the United States, China, Canada, and Brazil, exhibited higher centrality in this field. The size of the annual cycle represents the number of publications, with the United States (*n* = 763), China (*n* = 586), and Italy (*n* = 402) the top 3 countries. The UK, United States, and Italy are more closely cooperating with other countries. The country cooperation map is presented in Figure 3C.

**Table 1:** Top 10 countries in published research on non-motor symptoms of PD from 2013 to 2022.

Country	Articles	Citations	Average article citations	H-index
USA	763	11,269	24	65
China	586	8,296	13.8	42
Italy	402	6,587	23	53
United Kingdom	387	5,804	30.5	56
Germany	386	4,751	20	48
Spain	269	3,415	21.8	44
Canada	198	5,836	53.5	34
South Korea	190	2022	11.8	26
France	175	2,222	22	38
Netherlands	165	2,368	25.7	35

### Analysing the most productive institutions in non-motor symptoms of PD

**Figure 4:**
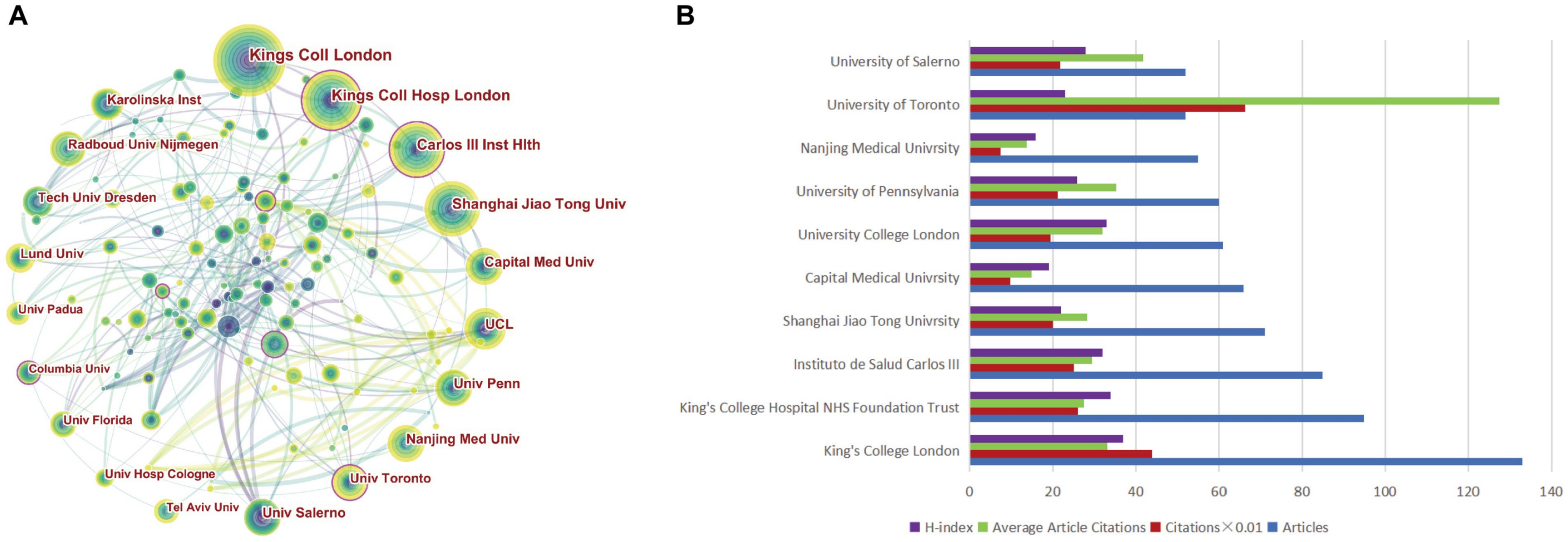
Visualization of institutions related to the research on non-motor symptom of PD in 2013–2022. **(A)** Occurrence of institutions; **(B)** The top 10 institutions were ranked based on the number of publications, total citations, average citations, and H-index.

A total of 4,292 institutions actively engaged in research investigating non-motor symptoms of PD. A collaborative map of institutions is presented in Figure 4A, with King’s College Hospital NHS Foundation Trust, *Instituto de Salud Carlos III*, and the University of Toronto (Toronto, Ontario, Canada) having high centrality, suggesting that these institutions publish at a high level and have a greater impact. The top 10 most productive institutions, with 3 each in China and the UK, and 1 each in the United States, Canada, Italy, and Spain, respectively (Table 2; Figure 4B). The University of London ranked first, with 133 cumulative publications, and had the highest H-index, with a high overall impact. The University of Toronto, with its highest citation frequency, had a high citation rate because its institutional publications are mostly reviews and guidelines.

**Table 2:** Top 10 publication institutions related to non-motor symptom of PD for 2013–2022.

Institution	Country	Articles	Citations	Average article citations	H-index
King’s College London	UK	133	4,404	33.11	37
King’s College Hospital NHS Foundation Trust	UK	95	2,618	27.55	34
Instituto de Salud Carlos III	Spain	85	2,509	29.51	32
Shanghai Jiao Tong University	China	71	2009	28.29	22
Capital Medical University	China	66	994	15.06	19
University College London	UK	61	1950	31.96	33
University of Pennsylvania	USA	60	2,118	35.3	26
Nanjing Medical University	China	55	755	13.72	16
University of Toronto	Canada	52	6,629	127.48	23
University of Salerno	Italy	52	2,180	41.92	28

### Analysing the high-influence authors in non-motor symptoms of PD

**Figure 5:**
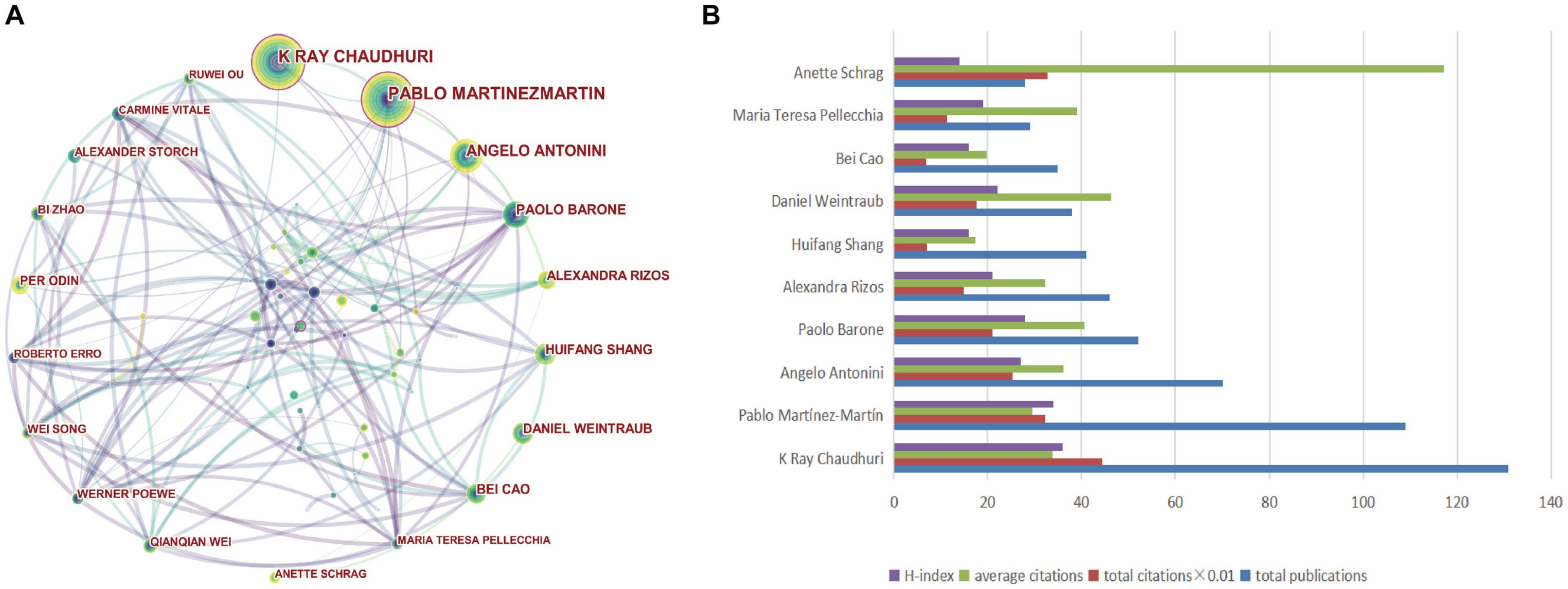
Visualization of authors and co-authorship related to the research on non-motor symptom of PD in 2013–2022. **(A)** Occurrence of authors; **(B)** The top 10 authors were ranked based on the number of publications, total citations, average citations, and H-index.

A total of 16,846 authors contributed to publications addressing non-motor symptoms of PD, as shown in the visualization map (Figure 5A). The evaluation criteria for the co-core authors included number of publications, total citations, and the H-index. The top 10 authors were all highly influential, with an H-index >14. Regarding specific authors, Chaudhuri (131 publications, 4,445 citations), Martínez-Martín (109 publications, 3,226 citations), and Antonini (70 publications, 2,531 citations) were the top 3 in terms of the number of publications and citations, respectively. The top 3 authors with the highest mean number of citations were Anette Schrag (117.21), Daniel Weintraub (46.34), and Paolo Barone (40.56). Pablo Martínez-Martín and Daniel Weintraub were from Spain and the United States, respectively, while the other authors were from China, Italy, and the UK. More information is available in Figure 5B and Table 3.

**Table 3:** Top 10 authors and co-cited authors related to non-motor symptom of PD for 2013–2022.

Author	Country	Organizations	Total publications	Total citations	Average citations	H-index
K Ray Chaudhuri	UK	King’s College Hospital	131	4,445	33.93	36
Pablo Martínez-Martín	Spain	Carlos III Institute of Health	109	3,226	29.6	34
Angelo Antonini	Italy	University of Padua	70	2,531	36.16	27
Paolo Barone	Italy	University of Salerno	52	2,109	40.56	28
Alexandra Rizos	UK	King’s College Hospital	46	1,486	32.3	21
Huifang Shang	China	Sichuan University	41	716	17.46	16
Daniel Weintraub	USA	University of Pennsylvania	38	1761	46.34	22
Bei Cao	China	Sichuan University	35	693	19.8	16
Maria Teresa Pellecchia	Italy	University of Salerno	29	1,134	39.1	19
Anette Schrag	UK	University College London	28	3,282	117.21	14

### Journals and disciplines distribution

A total of 637 journals published articles addressing the non-motor symptoms of PD, of which 124 had >5 articles. Among the top 10 journals (Table 4), *Parkinsonism & Related Disorders* topped the list, with a total output of 252 articles and 7,210 total citations, followed by *Movement Disorders* and *Frontiers in Neurology*, with 145 and 138 related publications, respectively. Of the top 10 journals, *Movement Disorders* had the highest impact factor (8.679) and the highest mean citation rate (52.42), suggesting that it may be the most popular journal. Overall, the number of journals was high, and these journals had impact factors between 3.2 and 8.679 in 2023.

**Table 4:** Top 10 journals and co-cited journals that published articles on non-motor symptoms of PD for 2013–2022.

Number	Journal	Total publications	Total citations	Average citation	H-index	IF
1	Parkinsonism & related disorders	252	7,210	28.61	45	4.1
2	Movement disorders	145	7,601	52.42	44	8.679
3	Frontiers in neurology	138	1,294	9.37	27	4.086
4	Journal of Parkinsons’ disease	137	1,688	12.32	27	5.52
5	Parkinson’s disease	99	895	9.04	27	3.2
6	Journal of neurology	96	2,121	22.09	25	6.682
7	Plos One	83	2,145	25.84	23	3.234
8	Journal of neural transmission	83	2,246	27.06	23	3.85
9	Journal of the neurological sciences	74	1,345	18.17	22	4.553
10	Neurological sciences	72	707	9.81	20	3.83

**Figure 6:**
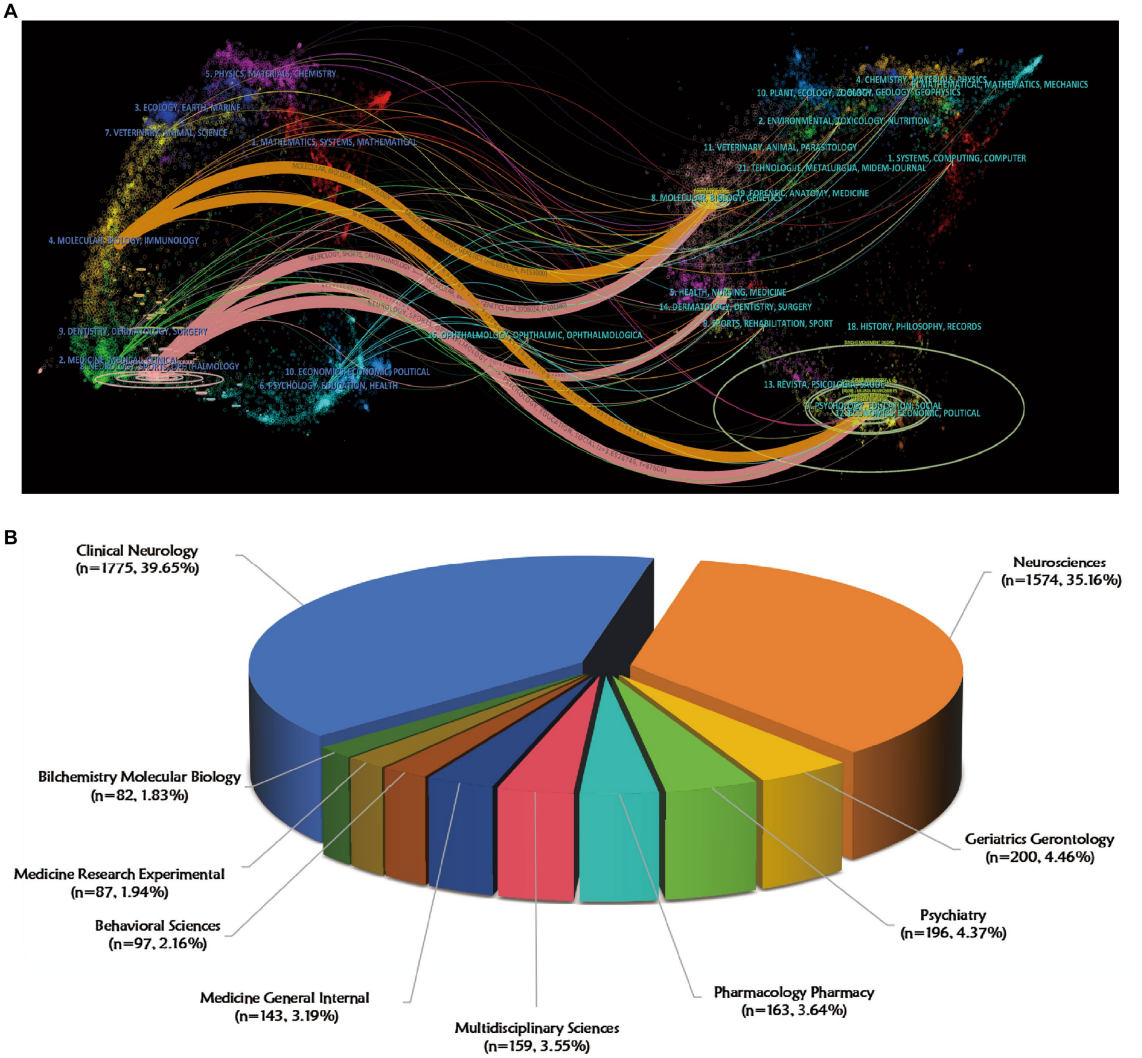
Visualization of journals and research fields on non-motor symptom of PD in 2013–2022. **(A)** A.dual-map overlay of journals that published literature on non-motor symptoms of PD. The ellipse in the figure represents the number of publications corresponding to a journal. The longer the horizontal axis of the ellipse is, the more authors are represented, and the longer the vertical axis of the ellipse is, the more papers are published on behalf of journals. **(B)** Top 10 research fields with the most published articles in non-motor symptom of PD.

The distribution of journals across fields, evolution of citation trajectories, and shifting of research centers can be illustrated using a dual-map overlay of journals (Chen and Leydesdorff, 2014; Zhang et al., 2021). In the dual-map overlay analysis (Figure 6A), citing journals are on the left and cited journals are on the right, with colored lines indicating the citation relationships. Results of analysis revealed that the cited journals related to non-motor symptoms in PD belonged mainly to the fields of molecular biology, immunology, neurology, sports, and ophthalmology, and were frequently cited by molecular biology, genetics, health, nursing, medicine, psychology, education, and sociology journals. Overall, the non-motor symptoms of PD were most closely related to research in basic science, neurology, and clinical and social topics. Further multidisciplinary alliances are recommended in the future. As shown in Figure 6B, clinical neurology was the most represented research area [*n* = 1775 records (39.65% of total articles)], followed by neuroscience [*n* = 1,574 (35.16%)], geriatric gerontology [*n* = 200 (4.46%)], and psychiatry [*n* = 196 (4.37%)].

### Research hotspots for non-motor symptoms of PD

**Figure 7:**
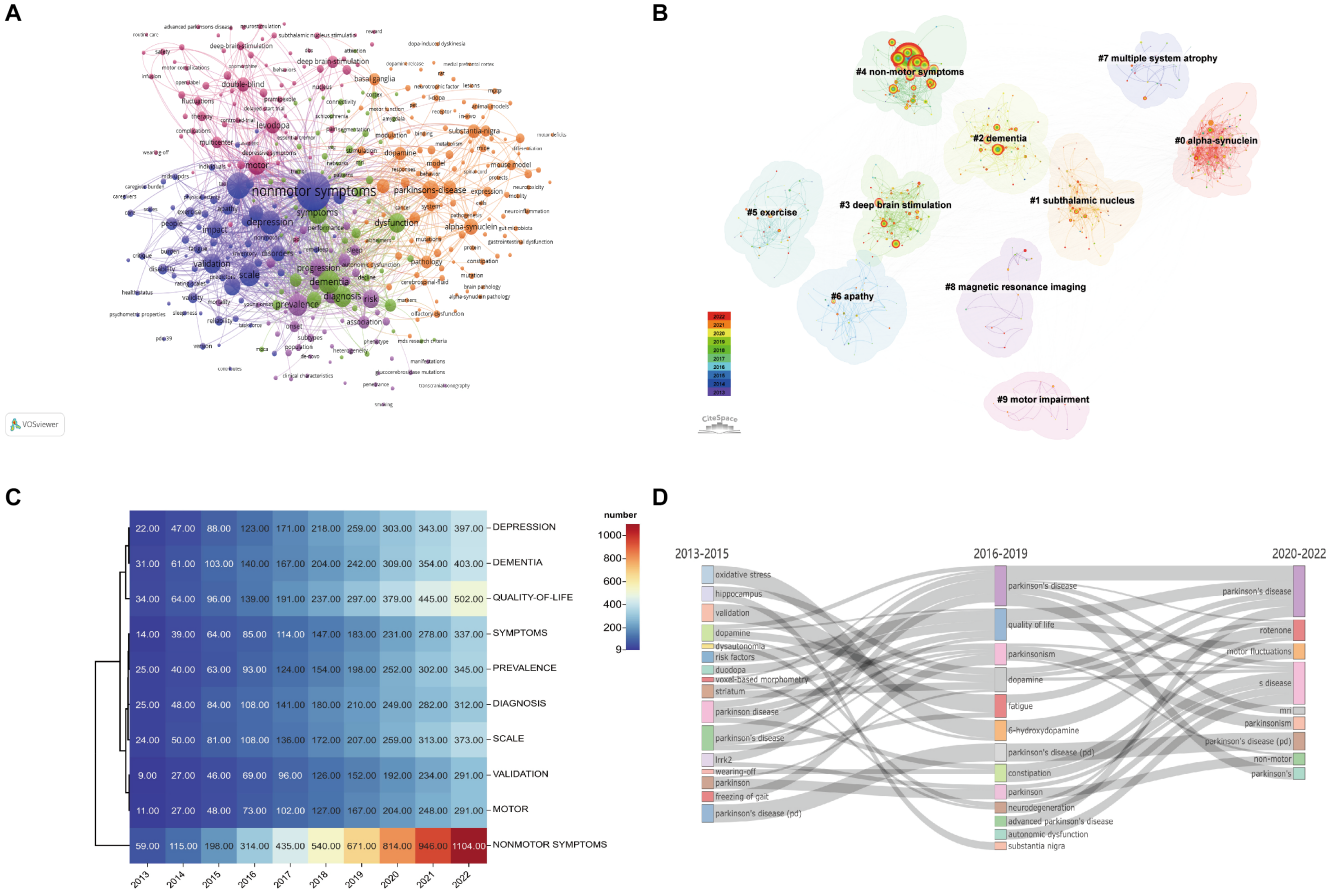
Analysis of keywords related to publications of non-motor symptoms in the PD field. **(A)** The co-occurrence network visualization map of keywords related to non-motor symptom in the PD field. The keywords were clustered into five groups according to their color. Large nodes represented keywords with high frequency; **(B)** High-frequency keywords cluster map of related literature. The nodes in the diagram represent references and the black font represents cluster labels. Nodes with the same color are located in the same cluster, which means they belong to the same cluster topic; **(C)** Heat map of the author’s keyword for publications published between 2013 and 2022. Data and visualization were generated from the documents menu of the Bibliometrix R-package. **(D)** Map of thematic evolution. The line alignment represents the evolution direction of the themes. Line width represents the number of keywords.

A total of 4,606 keywords were extracted from the publications, of which 389 appeared >10 times. Non-motor symptoms were at the core of all keywords, with the highest frequency (1,111 occurrences) and broadest association with other keywords (total link strength [TLS] 4,893), indicating that they have received attention in relevant research investigating PD (Figure 7A).

A keyword-clustering graph focuses on the structural features between clusters, highlighting the key points and important connections (Qin et al., 2020). Based on co-occurrence analysis, keywords were clustered using log-likelihood ratio (LLR) to create a keyword-clustering network graph (Figure 7B). The top 10 clustered tag groups (#0–9) examined and screened using clustered keywords represented a general framework for research on non-motor symptoms in PD (Table 5). Among the 8 keyword clusters, the research topics can be classified into three categories: #0, #7, and #8 are the first category, which mainly include pathological changes and testing methods; #2, #4, #6, and #9 are the second category, which mainly include clinical symptoms; and #1, #3, and #5 are the third category, which mainly include relevant treatments. Among these clusters, #0 α-syn contains most of the nodes, implying that α-synuclein (α-syn) pathological alterations have a significant impact on non-motor symptoms in PD.

**Table 5:** List of keywords cluster labels of non-motor symptoms in PD for 2013–2022.

Cluster-ID	Size	Silhouette	Cluster name	LLR
#0	136	0.641	Alpha-synuclein	Oxidative stress; rotenone; quality of life and MPTP
#1	80	0.615	Subthalamic nucleus	Functional connectivity; deep brain stimulation; cortex and basal ganglia
#2	80	0.681	Dementia	Cognitive impairment; progression; impairment and rem sleep behavior disorder
#3	76	0.715	Deep brain stimulation	Levodopa; motor fluctuations; safinamide and dyskinesia
#4	74	0.716	Non-motor symptoms	Quality of life; Parkinson’s disease; fatigue and health-related quality of life
#5	48	0.633	Exercise	Physical activity; rehabilitation; gait and telemedicine
#6	47	0.727	Apathy	Neuropsychiatric symptoms; diagnostic criteria; mild cognitive impairment and neural regeneration
#7	23	0.858	Multiple system atrophy	Autonomic nervous system; motor and non-motor symptoms; imaging and vagus nerve
#8	18	0.881	Magnetic resonance imaging	Machine learning; Parkinson’s disease; gait analysis and accuracy
#9	16	0.892	Motor impairment	Cognitive deficits; scopa-sleep; circadian entrainment and scales for outcomes in Parkinson’s disease-sleep

The keyword heat map revealed the frequency of keyword appearances in different years (Figure 7C). The keywords of authors in articles, published between 2013 and 2022, were investigated and the evolution of the top 10 keywords is presented in the Figure. By 2022, keywords related to PD had the highest frequency of non-motor symptoms (*n* = 1,104), followed by quality of life (*n* = 502), dementia (*n* = 403), and depression (*n* = 397).

“Keyword Plus” is a term that often appears in the title of publication references, is extracted automatically by the Web of Science through a computer algorithm, and has been confirmed by a broader descriptive (Qin et al., 2020). Therefore, it is appropriate and objective to use Keyword Plus to examine changes in research trends over time. A thematic evolution diagram summarizing various evolutionary associations using keywords (Figure 7D) demonstrated the development of non-motor symptoms in PD. To better reflect the research focus and trends, publications over the past 10 years were divided into three periods: 2013–2015, the first period of research mainly focused on exploring mechanisms such as oxidative stress, dopamine, and leucine-rich repeat kinase 2 (IRRK2); 2016–2019, the second period of research mainly focused on non-motor symptoms, such as quality of life, fatigue, constipation, and autonomic symptoms; and 2020–2022, the third period focused on motor fluctuations, MRI, and Parkinson’s syndrome.

### Highly cited references on non-motor symptoms of PD

**Figure 8:**
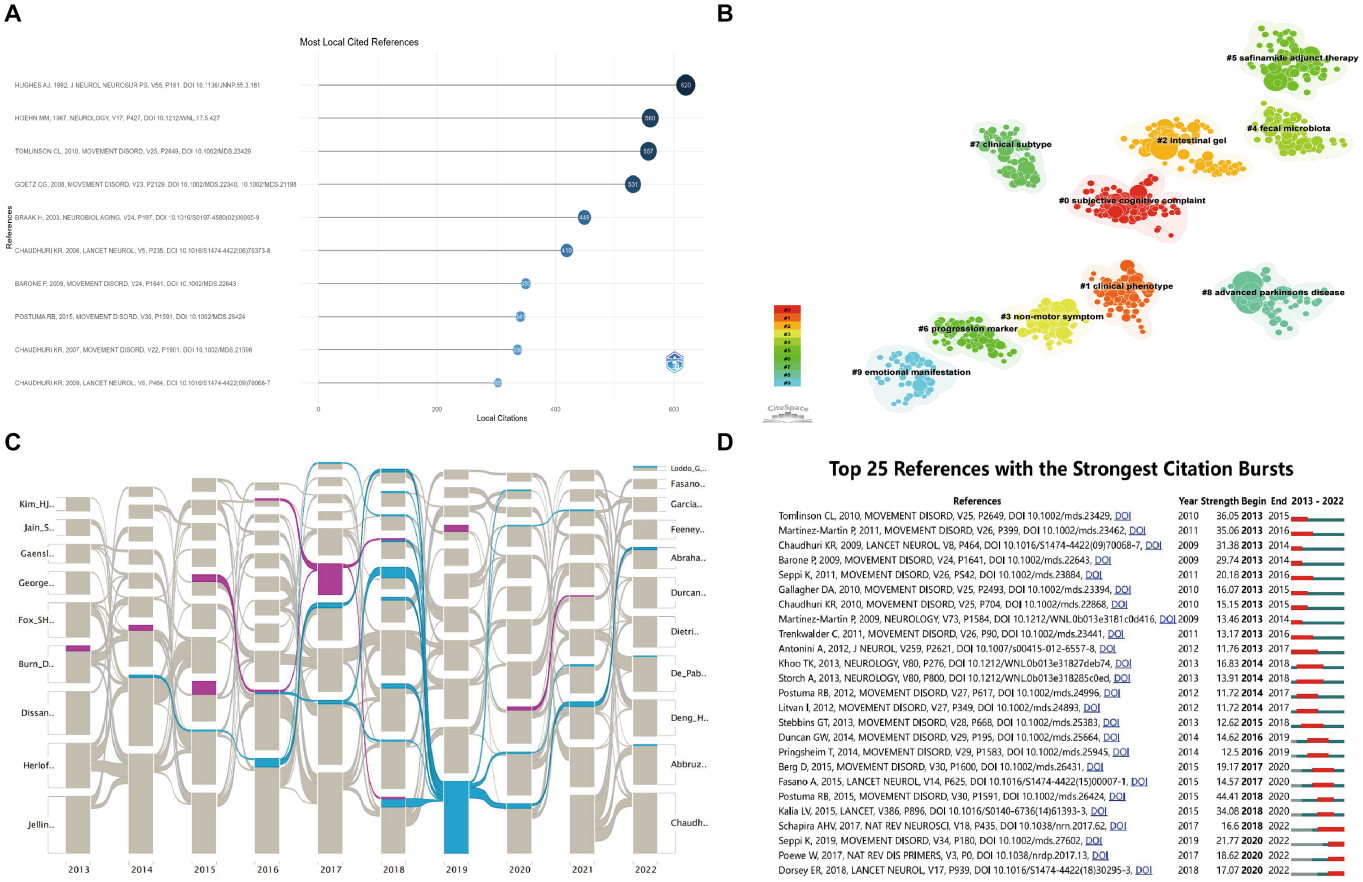
Analysis of co-citation references related to publications of non-motor symptom in the PD field. **(A)** Trends in co-cited references development over the 10 years. The blue line indicates the timeline of keywords, and the bubble size indicates the frequency of keywords; **(B)** The clustering map of relevant references. A total of 10 categories of references were obtained. The different color blocks represent different reference clusters; **(C)** Alluvial flow map of co-cited references in 10 years. Each line represented a study, and colored and continuous lines referred to articles that had been cited more than 8 years in 10 years; **(D)** Top 25 keywords with the strongest Co-occurrence frequency burst. The green bars represent the period when the keywords appear; the red bars represent the time interval when the keywords are found to erupt, indicating the beginning year, end year, and duration of the outbreak.

In total, 74,805 references were extracted, of which 695 were cited >20 times. Based on citation analysis of the literature reflecting these citations, the top 10 highly cited articles are listed in Figure 8A (detailed information is summarized Table 5), with number of citations ranging from 303 to 620, of which 4 articles were cited >500 times. Analyses of these 4 studies revealed that they directly addressed PD diagnosis, pathology, and treatment.

Ten sets of cluster labels (#0–9) were generated after cluster analysis of the cited literature using LLR (Figure 8B). The largest cluster was subjective cognitive complaint, followed by clinical phenotype, intestinal gel, non-motor symptoms, fecal microbiota, safinamide adjunct therapy, progression marker, clinical subtype, advanced PD, and emotional manifestations.

The alluvial plot (Figure 8C) depicts the most frequently cited articles over the past 10 years. Two of them (Stocchi et al., 2014; Amami et al., 2015) were cited for more than 8 years from to 2013–2022, one related to wearing-off for early diagnosis of PD and the other related to subthalamic deep brain stimulation for improvement of impulse control behaviors in PD.

Identifying references with the strongest citation explosions may help researchers determine popular topics and changes in research directions that are becoming increasingly popular in the field (Figure 8D). References with the strongest citation bursts were examined, and the top 3 strongest bursts had burst intensities of 44.41 (Postuma et al., 2015), 36.05 (Tomlinson et al., 2010) and 35.06 (Martinez-Martin et al., 2011). In addition, Citespace identifies influential references over time. Articles published around 2013 focused on topics such as “nonmotor symptoms,” “health-related quality of life,” and “non-motor symptoms scale.” Publications around 2017 focused on topics such as “pre-motor disorders in Parkinson’s disease,” “rapid eye movement (REM) sleep behavior disorder (RBD),” “constipation,” “depression,” and “cognitive.” Publications around 2022 focused on topics such as “early diagnosis biomarkers,” novel MRI techniques,” and “deep brain stimulation.”

## Discussion

PD is among the most common progressive neurodegenerative disorders and characterized by the progressive loss of dopaminergic neurons in the substantia nigra and basal ganglia. Lewy bodies, associated with progressive dopaminergic neuronal death, and the presence of distinctive granular α-synuclein aggregates in spherical pale bodies, are the hallmark neuropathological features of PD (Goiran et al., 2022). PD is typically recognized for its major motor symptoms, such as resting tremor, muscle rigidity, bradykinesia, and postural instability (Tansey et al., 2022). Additionally, non-motor symptoms include olfactory dysfunction (Gu et al., 2024), RBD (Huang B. et al., 2023), depression (Ahmad et al., 2023), cognitive disorders (Siciliano et al., 2023), psychiatric (Weintraub et al., 2022), and insomnia (Savica et al., 2009). Such conditions may occur 10–20 years before the onset of dyskinesia in patients with PD and can persist during disease progression, heavily detracting from patient quality of life and incurring high costs (Munhoz et al., 2015; Leite Silva et al., 2023). As such, the study of non-motor symptoms of PD has increased yearly as a focus of attention in the past decade.

Bibliometric analyses and mapping have developed rapidly in recent years as the scientific community has become increasingly interested in the results of various bibliometric analyses (Donthu et al., 2021). By focusing on the current state and trends in global research, the present study explored the hotspots and frontiers of research investigating non-motor symptoms of PD. Despite the scope of research in this area, there is relative confusion and a lack of analysis of research priorities and trends. To our knowledge, this is the first bibliometric analysis to identify emerging trends and hotspots in research investigating the non-motor symptoms of PD.

### Trends in the publication of research on non-motor symptoms of PD

Over the past 10 years, there has been a relatively steady increase in the number of studies published annually in the international research literature on non-motor symptoms of PD. This increasing trend suggests that an increasing number of investigators are focusing their research on PD non-motor symptoms. However, the large increase in publications has led to a decline in the average number of citations per year, and the field should continue to explore new directions to increase its influence. From the global impact analysis, the United States had the most publications in the field of PD non-motor symptoms, with significant contributions from one of its prestigious universities, the University of Pennsylvania (Philadelphia, PA), and Daniel Weintraub as Consultant Psychiatrist, Parkinson’s Disease and Movement Disorders Center (PDMDC), Hospital of the University of Pennsylvania. Philadelphia has published a large number of high-level articles in the field of PD non-motor symptoms. A recent publication in the *British Medical Journal* (Weintraub et al., 2022) summarizes the epidemiology and neurobiology of the management strategies for psychiatric and cognitive complications in PD, and suggests that in the future, new therapeutic strategies should be developed to improve psychiatric and cognitive deficits in patients with PD and improve quality of life. With the increase in the number of patients with PD each year, several institutions specializing in the study of PD have emerged. Among them, King’s College (London, UK) has published the most research on non-motor symptoms of PD in a decade, and its H-index is also ranked number 1, which indicates that its overall research level is high. The University of Toronto (Toronto, Ontario, Canada), although ranked ninth in the number of articles published, ranked first in the number of citations, suggesting that this institution publishes more authoritative articles and is an outstanding contributor to the study of non-motor symptoms in PD. The major contributor was Lorraine Kalia, with a highly cited article on PD in *The Lancet*, describing the early onset of non-motor symptoms in PD and the challenges of symptom management in later life (Kalia and Lang, 2015).

Regarding outstanding contributors to the field of research investigating non-motor symptoms in PD, K Ray Chaudhuri from the Department of Clinical Neurology, University of London, England, is a major contributor to the field, with the most publications, total citations, and H-index, suggesting that his team has been more prominent in the research on non-motor symptoms in PD over the past decade. Publications with high citations published in *Lancet Neurology* in 2006 (n = 1818) (Chaudhuri et al., 2006) and 2009 (n = 1,124) (Chaudhuri and Schapira, 2009) examined the dopaminergic neuronal basis for the occurrence of non-motor symptoms such as depression, apathy, SEM, and erectile dysfunction in PD, summarized the diagnosis and management of the non-motor symptoms of PD, and discussed evidence that these symptoms are, at least in part, treatable with various dopaminergic strategies. Anette Schrag, also from the Department of Clinical Neurology at the University of London, England, published an article with the highest average number of citations, although it ranked 10th in terms of the total number of articles published. This indicates higher quality articles with a higher number of citations. Principally, the 2006 publication of the Movement Disorder Society-sponsored revision of the unified Parkinson’s Disease Rating Scale (MDS-UPDRS) (Goetz et al., 2007) revision in *Movement Disorders* and the publication of the results of the clinical evaluation of the MDS-UPDRS revision the following year, revealed that the clinical results support the validity of the MDS-UPDRS for rating PD (Goetz et al., 2008).

### Research findings on non-motor symptoms of PD

Based on a citation frequency analysis of the literature, the study by Poewe et al. (2017) in 2017, published in *Nature Reviews Disease Primers*, elicited a high number of citations (*n* = 2,498). This illustrates that non-motor symptoms of PD are as important as movement disorders, and that non-motor symptoms increase overall disability. The potential molecular pathogenesis and pathways of PD are related to a-synuclein proteostasis, mitochondrial function, oxidative stress, calcium homeostasis, and neuroinflammation. The challenge in the future is to identify markers of prodromal disease staging and develop new disease-modifying therapies. Also frequently cited in the literature is a prospective cohort study on movement disorders by the team of Scheperjans and Filip published in 2015 (Scheperjans et al., 2015). The development of PD may be associated with altered flora abundance, in which the abundance of Prevotellaceae decreased by 77.6% in patients with PD compared to that in controls. However, decreased Prevotellaceae abundance alone lacks specificity for diagnosing PD. The temporal and causal relationship between the gut microbiota and PD, as well as the suitability of gut microbiota as a biomarker, should be further explored in the future.

Furthermore, a prospective cohort study by Fereshtehnejad and Seyed-Mohammad was published in *JAMA Neurology* (Fereshtehnejad et al., 2015) aimed to redefine the clinical subtypes of patients with PD, in which patients with the diffuse/malignant phenotype, despite similar age and disease duration, are more likely to present with mild cognitive impairment, upright hypotension, and RBD, and to have faster progression of cognitive impairment compared with other non-motor symptoms at follow-up. Therefore, screening patients with mild cognitive impairment, upright hypotension, and RBD identified the diffuse/malignant subgroup of patients with diffuse/malignant PD, which is expected to progress at the fastest rate.

### Research hotspots and frontiers

Based on visualization results, such as keyword clustering maps, keyword heat maps, and reference burst maps, research trends and hot topics in the field can be identified. Keywords, including “non-motor symptoms,” “quality of life,” “cognition,” and “depression,” have been the focus of PD research for the past decade. The pathophysiology of PD suggests that neurotoxic α-synuclein accumulates in large quantities in the substantia nigra of the midbrain to form Lewy vesicles (Campos-Acuña et al., 2019), defects in dopaminergic synthesis by modulating neuronal membrane stability, affecting presynaptic signaling and transmembrane vesicular transport (Hansson, 2021). Dopamine deficiency, in addition to motor symptoms and some non-motor signs and symptoms, precedes motor symptoms by several years (Leite Silva et al., 2023). While the diagnosis of PD often relies on motor symptoms, it is particularly important to achieve an early diagnosis and treatment through the recognition of early non-motor symptoms to reduce the progression of this neurodegenerative disease (Schapira et al., 2017). Quality of life has been demonstrated to have good reliability, validity, responsiveness, and reproducibility in the assessment of patients with PD (Peto et al., 1995).

Depression is the most common non-motor symptom in all stages of PD. The impact of depression on quality of life is more significant and serious than that of other motor and non-motor symptoms. Episodes of depression-like symptoms sometimes precede motor symptoms and become severe in the later stages of PD progression, leading to severe disability and impaired quality of life (Ffytche et al., 2017). Neurotransmitter dysregulation (Yadav and Kumar, 2022), diminished nutritional support (Rahmani et al., 2019), hypothalamic–pituitary–adrenal axis disruption (Hemmerle et al., 2014) and neuroinflammation (Pessoa Rocha et al., 2014) are potential neurobiological mechanisms underlying depression in PD. Consequently, new therapeutic approaches targeting these molecular mechanisms must be developed to alleviate depression in patients with PD (Ahmad et al., 2023). In addition, cognitive impairment is 6 times more prevalent in patients with PD than in healthy individuals (Aarsland et al., 2001). It is one of the most important non-motor manifestations of PD, which can severely affect quality of life and functioning, and has been shown to have realistic economic consequences beyond motor symptoms (Leroi et al., 2012; Chandler et al., 2021).

An analysis of the literature suggests that mild cognitive impairment (MCI) is a common non-motor symptom of PD, with up to 30% of patients without dementia meeting the criteria for MCI (PD-MCI) (Litvan et al., 2012). In a study involving patients with PD with normal cognitive function, PD-MCI occurred in nearly 50% of patients within 6 years, and all patients who developed PD-MCI subsequently progressed to dementia within 5 years (Pigott et al., 2015). Subjective cognitive complaints are sensitive indicators for assessing PD-MCI (Hong et al., 2014; Purri et al., 2020). Consequently, subjective cognitive complaints have become a popular topic in PD research in recent years. Clinical phenotype is another hotspot of research on the non-motor symptoms of PD, and the phenotype of PD can be divided into the motor phenotype, with tremor as the dominant symptom, including gait disturbance, motor inability, and rigidity (Schiess et al., 2000). The non-motor phenotype includes cognitive impairment, depression, RBD, severe loss of olfaction, and urinary dysfunction (Zis et al., 2015). Clinically, PD is highly heterogeneous, making subtyping a useful strategy for guiding research and clinical practice (Marras and Chaudhuri, 2016).

In addition, PD is a manageable neurodegenerative disease with a disease-induced disability that worsens over time. Identifying PD biomarkers to enable early diagnosis can improve treatment outcomes and prognosis (Parnetti et al., 2019). Publications addressing this topic have proliferated, and early diagnosis and identification of biomarkers have become the focus of research in recent years. Regarding biomarkers of PD, the formation of Lewy bodies and neural inclusions in the form of Lewy neuromasts by aggregation and misfolding of α-syn are the main pathological products of PD. As diagnostic biomarkers continue to progress, α-syn in new tissue and fluid specimens such as cerebrospinal fluid (Shahnawaz et al., 2017), blood (Abd-Elhadi et al., 2015), salivary glands (Vilas et al., 2016), and skin (Donadio et al., 2014; Wang et al., 2021) may serve as biomarkers of the prodromal disease stage (Tolosa et al., 2021). Novel MRI techniques, including neuromelanin imaging, quantitative susceptibility mapping (QSM), and visual assessment of dorsal nigrostriatal high signals, have the potential to assess nigrostriatal pathology in PD and have been a major focus of recent research efforts (Murakami et al., 2015; Wang et al., 2019). However, these novel MRI techniques are poor at distinguishing PD from other types of degenerative Parkinsonian syndromes because nigral pathology is common to all of these. Recent reports have also suggested a high discriminative accuracy between PD and multiple system atrophy and progressive supranuclear palsy using observer-independent machine learning approaches with automated volumetry, automated voxel-based diffusivity (Murakami et al., 2015; Scherfler et al., 2016) or multimodal MRI combining several magnetic resonance parameters (Péran et al., 2018).

Although conventional therapeutic drugs aim to restore the depleted dopamine levels in the brain, the development of PD involves multiple interacting neurotransmitters. Targeting non-dopamine systems may be an alternative approach (Fox, 2013). The novel dual mechanism of action of safinamide in recent years, dopaminergic (reversible monoamine oxidase-B inhibition) and non-dopaminergic (modulation of aberrant glutamate release), provides a unique approach for treating both motor and non-motor symptoms (Müller and Foley, 2017). Safinamide, as an add-on to levodopa, significantly reduces OFF time and increases ON time in PD patients with “on–off phenomena,” and improves non-motor symptoms and quality of life in those with PD (Hattori et al., 2020; Wei et al., 2022). Safinamide adjunct therapy has been increasingly reported for non-motor symptoms of PD in the past decade and is the focus of scholarly attention. As PD progresses, patients experience worse quality of life due to fluctuating drug responses, levodopa-naïve tremors, and drug-related complications (e.g., levodopa-induced dyskinesia) (Turcano et al., 2018; Verschuur et al., 2019), Deep brain stimulation (DBS) can better improve the functioning and quality of life of such patients, which is continuously being investigated by researchers in this direction (Weaver et al., 2009). Despite increasing research, continuous DBS (cDBS) may induce side effects such as dysarthria and imbalance, which may be related to overstimulation and abnormal circuits (Limousin and Foltynie, 2019; Tanaka et al., 2021). To overcome these shortcomings, novel adaptive DBS (aDBS), an emerging field, recognizes input signals associated with changes in patient symptoms, such as brain or motor signals, to provide responsive, optimized stimulation in real-time (Guidetti et al., 2021). Its benefits have been demonstrated in several animal experiments (Johnson et al., 2016) and clinical studies (Little et al., 2016), potentially surpassing those of conventional cDBS as a therapeutic option for non-motor symptoms in PD (Wang et al., 2023).

## Conclusion

This study used bibliometric and visualization techniques to provide a concise overview and assessment of the global state of research, trends, prominent avenues of research, and the emerging focus on PD non-motor symptoms. Our bibliometric study revealed a steadily increasing trend in the number of articles addressing non-motor symptoms in PD, suggesting that these symptoms are an important and urgent research topic. We identified major contributors, publications, and geographical regions within the research field. The greatest number and impact of articles was published in the United States. Cognitive deficits, depression, SEM, and olfactory deficits among the non-motor symptoms of PD are the focus of the current research. Early diagnosis is necessary for treatment and prognosis, and the screening of biomarkers and development of novel MRI techniques are directions for future research. For the treatment of non-motor symptoms of PD, pharmacological treatment using safinamide can significantly improve non-motor symptoms in patients with PD and improve quality of life; moreover, this new type of non-dopaminergic neurotransmitter may be a new direction for the treatment of PD in the future. In addition, non-pharmacological novel aDBS, as an emerging field to compensate for the side effects produced by DBS alone, providing responsive and optimized stimulation in real-time according to brain signals, may be a therapeutic option for the non-motor symptoms of PD. In summary, current research hotpots are the screening of biomarkers for non-motor symptoms of PD, the development of fMRI and novel aDBS techniques, and the development of safinamide drugs. With increased research on non-motor symptoms of PD, PD is increasingly viewed as a multi-organ, multi-system pathology that arises from the interaction of susceptible genetic factors with challenging environmental interactions during aging-related decline. Thus, the full contemplation of PD and the PD patient may indicate new paths to follow.

## Strength and limitations

The present study provides the first comprehensive, objective, and intuitive analysis of publications addressing the non-motor symptoms of PD and their trends, which can provide reference for researchers investigating this topic. In addition, we used various bibliometric software packages to investigate the different dimensions of research hotspots, enabling us to obtain more accurate and objective findings. This study, however, had some limitations, the first of which was only the inclusion of original articles and reviews published in English from the WoSCC database, which may differ slightly from actual results. Furthermore, the continuous update of the database had a subtle impact on the results of the analyses, and more studies need to be included for future refinement. Furthermore, this study included only original research and reviews, the language was limited to English, and the period was limited to the past 10 years of research, which may have reduced the number of publications retrieved and led to the omission of some publications. Another limitation of this study was the inability of the bibliometric software to distinguish between articles focusing on human and animal models. Finally, a few authors in the data may have duplicate names, and some may have held honorary or part-time positions at different universities. Nevertheless, we believe that this study accurately describes the current state of research and general trends regarding this topic. This study may lay the groundwork for neuroscientists and related researchers to quickly identify research priorities and emerging research trends investigating the non-motor symptoms of PD.

## Author contributions

XL: Writing – original draft. CC: Formal analysis, Writing – original draft. TP: Data curation, Investigation, Writing – original draft. XZ: Formal analysis, Methodology, Writing – original draft. XS: Funding acquisition, Resources, Writing – original draft. ZZ: Resources, Supervision, Writing – original draft. DW: Supervision, Writing – review & editing. XC: Validation, Writing – review & editing.

## Footnotes

^1^https://www.graphica.app/

^2^https://chiplot.online
